# Biochemical, Nutritional, and Clinical Parameters of Vitamin B12 Deficiency in Infants: A Systematic Review and Analysis of 292 Cases Published between 1962 and 2022

**DOI:** 10.3390/nu15234960

**Published:** 2023-11-29

**Authors:** Miriam Wirthensohn, Susanne Wehrli, Ulf Wike Ljungblad, Martina Huemer

**Affiliations:** 1 Department of Pediatrics, Landeskrankenhaus Bregenz, 6900 Bregenz, Austria; 2 Department of Psychosomatics and Psychiatry, University Children’s Hospital, University of Zurich, 8032 Zurich, Switzerland; 3 Division of Child and Adolescent Health Psychology, Department of Psychology, University of Zurich, 8050 Zurich, Switzerland; 4 Children’s Research Centre, University Children’s Hospital Zurich, University of Zurich, 8032 Zurich, Switzerland; 5 University Research Priority Program “ITINERARE—Innovative Therapies in Rare Diseases”, University of Zurich, 8032 Zurich, Switzerland; 6 Department of Pediatrics, Vestfold Hospital Trust, NO-3168 Tønsberg, Norway; 7 Division of Metabolism and Children’s Research Center, University Children’s Hospital Zurich, University of Zurich, 8032 Zurich, Switzerland; 8 Vorarlberg University of Applied Sciences, Competence Area Healthcare and Nursing, 6850 Dornbirn, Austria

**Keywords:** newborn screening, breastfeeding, methylmalonic acid, homocysteine

## Abstract

Pooled data from published reports on infants with clinically diagnosed vitamin B12 (B12) deficiency were analyzed with the purpose of describing the presentation, diagnostic approaches, and risk factors for the condition to inform prevention strategies. An electronic (PubMed database) and manual literature search following the PRISMA approach was conducted (preregistration with the Open Science Framework, accessed on 15 February 2023). Data were described and analyzed using correlation analyses, Chi-square tests, ANOVAs, and regression analyses, and 102 publications (292 cases) were analyzed. The mean age at first symptoms (anemia, various neurological symptoms) was four months; the mean time to diagnosis was 2.6 months. Maternal B12 at diagnosis, exclusive breastfeeding, and a maternal diet low in B12 predicted infant B12, methylmalonic acid, and total homocysteine. Infant B12 deficiency is still not easily diagnosed. Methylmalonic acid and total homocysteine are useful diagnostic parameters in addition to B12 levels. Since maternal B12 status predicts infant B12 status, it would probably be advantageous to target women in early pregnancy or even preconceptionally to prevent infant B12 deficiency, rather than to rely on newborn screening that often does not reliably identify high-risk children.

## 1. Introduction

Vitamin B12 (B12) or cobalamin (Cbl) is a water-soluble vitamin exclusively contained in foods from animal sources, such as milk, eggs, meat, and fish. In infants, persistent, severe B12 deficiency causes potentially irreversible, mainly neurological symptoms, often accompanied by anemia [1,2,3]. Recommended dietary intake of B12 is 0.5 µg/d for infants in the first three months, 1.4 µg/d from 4–12 months, 4 µg/d for adults, 4.5 µg/d during pregnancy, and 5.5 µg/d for breastfeeding women [4]. The mechanisms of maternofetal B12 transfer are not completely known [5,6,7], but transport seems facilitated by transcobalamin and in favor of the fetus. Approximately 70% of B12 transported across the placenta is bound to transcobalamin in contrast to only about 30% in maternal blood [5], and higher placental transcobalamin concentrations correlate with higher cord-blood B12 levels [7].

Maternal B12 deficiency (due to, e.g., a vegan diet or B12 malabsorption) leads to limited intrauterine supply, resulting in low neonatal B12 stores. Since females with B12 deficiency also have low B12 in breast milk, breastfed infants may not replenish their B12 stores sufficiently [8,9].

Holo-transcobalamin (holo-TC) is B12 bound to transcobalamin II. Holo-TC is the fraction of B12 able to enter cells. Methylmalonic acid (MMA) and total homocysteine (tHcy) are markers for functionally relevant, intracellular B12 deficiency, and methionine may also be informative. Intracellularly, B12 is involved in two reactions. Adenosylcobalamin is a cofactor for the enzyme methylmalonyl-CoA mutase that catalyzes the reversible isomerization of methylmalonyl-CoA to succinyl-CoA in mitochondria. Adenosylcobalamin deficiency results in the accumulation of MMA. In the cytosol, methylcobalamin is a cofactor for the interacting enzymes methionine synthase reductase and methionine synthase and the remethylation of homocysteine (Hcy) to methionine. Methylcobalamin deficiency results in the accumulation of Hcy; methionine levels may be normal or low [3,10]. 

Clinical signs of infant B12 deficiency typically develop within the first months of life. Developmental arrest or regression, irritability, feeding difficulties, seizures, movement disorders, apathy, anemia, and brain atrophy are characteristic. Irreversible damage may occur if the condition is not diagnosed and treated timely with B12 [1,2,3].

This study reviews and analyses pooled data from published case reports and case series reporting biochemical and clinical information on infants clinically diagnosed with B12 deficiency. The study aims to describe and analyze the clinical presentation, risk and protective factors, and diagnostic strategies targeting infant B12 deficiency. The purpose of this study is to raise awareness for this treatable condition to achieve early diagnosis; to identify effective diagnostic strategies, and to inform screening and prevention strategies. 

## 2. Materials and Methods

### 2.1. Literature Search

The systematic review procedure was conducted according to the PRISMA guidelines [11]. The project was registered with the Open Science Framework (https://osf.io/drzb8/?view_only=c1fb99fba7a7469dbeb8ce5af43fd2d6 accessed on 15 February 2023) before data analysis.

Clinical and biochemical parameters of interest were defined before the literature search based on textbook knowledge and recent reviews [3,5]. Clinical signs newly mentioned in the selected publications were added to the predefined set and searched for in all the included publications.

**Figure 1:**
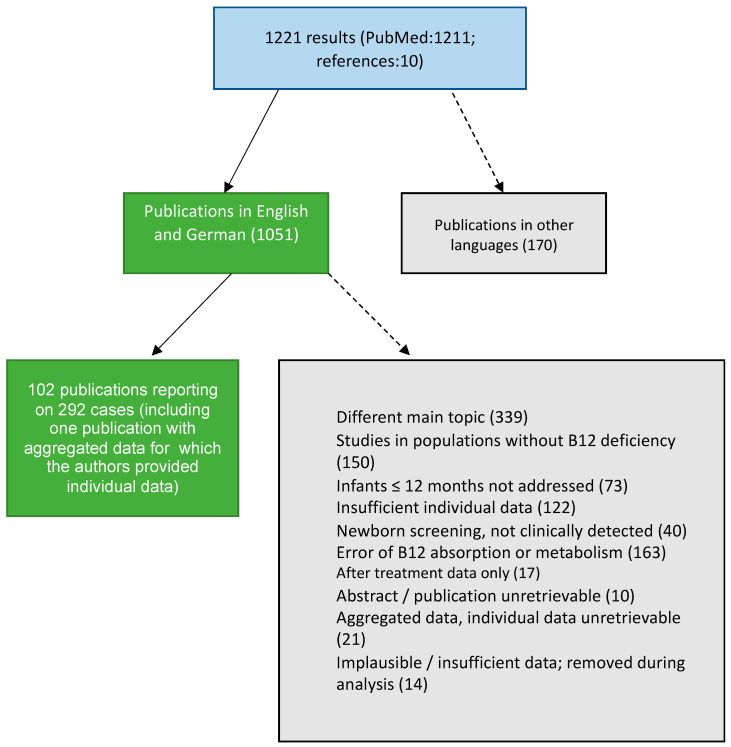
Identification and selection of publications following PRISMA and The JBI Critical Appraisal Checklist for Case Reports [11,12].

The electronic literature search of the PubMed database was conducted between 31 August and 31 December 2022, using the following search terms: (vitamin B12 OR cobalamin OR vitamin B12 deficiency OR cobalamin deficiency) AND (children OR infant OR infancy) filtered by language (English AND German). No geographical or publication date-related filters were applied. Detected studies were manually selected by two investigators (MW and MH) by reviewing the abstract and, if necessary, the full text of the article. Reference lists of included publications were manually searched for further eligible studies. Studies not meeting the inclusion criteria “clinical diagnosis of infant B12 deficiency, individual biochemical and clinical data reported”, or The JBI Critical Appraisal Checklist for Case Reports [12] criteria were excluded. Twenty-two reports included aggregated data, and the respective corresponding authors were contacted via e-mail and invited to provide individual data (successful for one publication) (Figure 1).

### 2.2. Data Extraction

Time and laboratory variables were recorded as numerical values. Clinical symptoms and dietary habits were recorded as present, not present, or unknown. 

The biomarkers of interest for infants and mothers were B12 (pmol/L), MMA in urine (mmol/mol creatinine), MMA in plasma (µmol/L), holo-TC (pmol/L), tHcy (µmol/L), and serum folate (nmol/L). 

The clinical parameters (infant) were sex, age at first symptoms, age at diagnosis, failure to thrive, irritability, seizures, anemia, cerebral atrophy, delayed myelination, enlarged ventricles, hypotonia, apathy/lethargy, vomiting, refusal of solid foods, developmental delay, and movement disorder. The nutritional parameters (infant) were breastfeeding only, partial breastfeeding, formula total, formula partial, solid foods mixed, solid foods vegetarian, and solid foods vegan. 

The maternal nutrition and health parameters were B12 malabsorption (intrinsic factor antibodies and antiparietal cell antibodies), vegan, vegetarian, mostly vegetarian, or mixed diet.

### 2.3. Statistical Methods

Analyses were performed using RStudio [13]. Biomarkers were log-transformed to obtain a distribution closer to the normal distribution. The analysis followed a stepwise approach. 

In step one, the characteristics of the included publications were analyzed according to how many studies were informed on the parameters. Furthermore, undetectable, implausible, or nonspecific values were identified.

Step two involved a descriptive analysis of the parameters of interest (calculation of means, ranges, standard deviations (SD), and frequency statistics) and documentation of variables that were not included in further analyses due to insufficient power. Infant and maternal biomarker distributions were examined graphically using violin plots that combined density and boxplots. 

In a third step, zero-order bivariate correlations between continuous variables were calculated to examine associations between infant biomarkers and medical data, nutritional parameters, and maternal biomarkers. Chi-square tests were calculated to examine associations between the co-occurrence of clinical outcomes. Groups were defined according to published categories of infant serum B12 levels (<148 pmol/L: probable deficiency; 148 to 258 pmol/L: possible deficiency; >258 pmol/L: unlikely deficiency) [14,15,16] and compared by chi-square tests and ANOVA for clinical outcomes, maternal B12 levels, and diet. 

In a fourth step, regression analyses were performed to identify potential risk factors for low B12 levels and negative clinical outcomes. Interaction and mediation effects between potential risk factors for low B12 levels and clinical outcomes were examined. Mediation effects were calculated using structural equation modeling. In all regression analyses, age at first symptoms, age at diagnosis, sex, and child serum folate level were used as control variables, except for the analyses in which age at first symptoms, age at diagnosis, and diagnostic delay were included as predictors.

## 3. Results 

### 3.1. Descriptive Statistics of Included Studies

A total of 116 studies were included in step one of the analyses. Fourteen publications covering 16 cases had to be excluded due to missing, implausible, or non-numeric data (e.g., ranges or interpretations of values) [17,18,19,20,21,22,23,24,25,26,27,28,29].

Of the remaining 102 studies that reported 292 cases [30,31,32,33,34,35,36,37,38,39,40,41,42,43,44,45,46,47,48,49,50,51,52,53,54,55,56,57,58,59,60,61,62,63,64,65,66,67,68,69,70,71,72,73,74,75,76,77,78,79,80,81,82,83,84,85,86,87,88,89,90,91,92,93,94,95,96,97,98,99,100,101,102,103,104,105,106,107,108,109,110,111,112,113,114,115,116,117,118,119,120,121,122,123,124,125,126,127,128,129,130,131], most originated from Asia (n = 41), followed by Europe (n = 36), North America (n = 15), Oceania (n = 7), Africa (n = 2), and South America (n = 1). Twenty-one publications were from Turkey, followed by the United States (n = 15) and India (n = 10). 

The earliest publication year was 1962, the latest 2022, with 25.5% (n = 26) published between 1962 and 1999, 23.5% (n = 24) between 2000 and 2009, and 51% (n = 52) between 2010 and 2022.

Infant B12 (n = 288 reports, 261 with numerical values) was most frequently reported, followed by infant tHcy (n = 155, 154 with numerical values), maternal B12 (n = 241, 239 with numerical values), and infant serum folate (n = 133, 111 with numerical values). MMA in urine was collected sporadically before and regularly from 2000 to 2010 (53%). From 2011, plasma MMA (43%) was favored over urine MMA (10%). tHcy was sporadically assessed before and measured in about 60% of cases since 2000. Holo-TC was assessed in only 21% of cases since 2011. Folate was frequently measured to address folate deficiency as an important differential diagnosis in anemia.

Regarding clinical parameters, information on their presence or absence in a case was provided most frequently for anemia (n = 268), followed by hypotonia (n = 172), developmental delay (n = 157), apathy/lethargy (n = 141), movement disorder (n = 131), failure to thrive (n = 103), irritability (n = 88), and cerebral atrophy (n = 81). Of the nutritional parameters, information on exclusive breastfeeding (n = 261) was reported most frequently, followed by information on partial breastfeeding (n = 184) and maternal diet (n = 206). 

### 3.2. Descriptive Statistics of Included Cases

**Figure 2:**
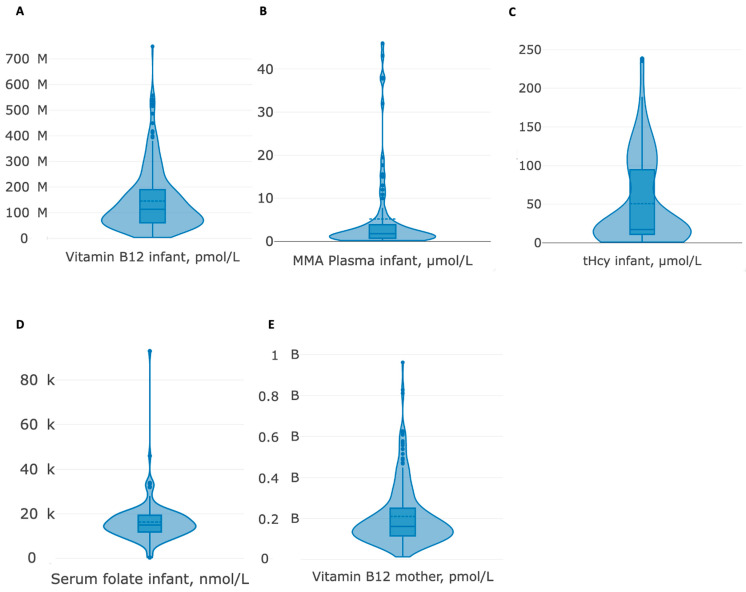
(**A**–**E**) Violin plots of numerical biomarkers.

**Figure 3:**
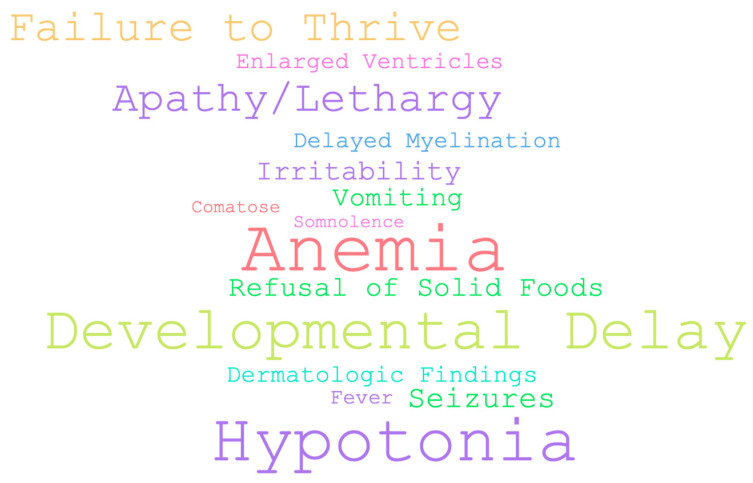
Clinical presentation: font size reflects the frequency of reported occurrence referring to the total number of entries on the respective parameter.

**Figure 4:**
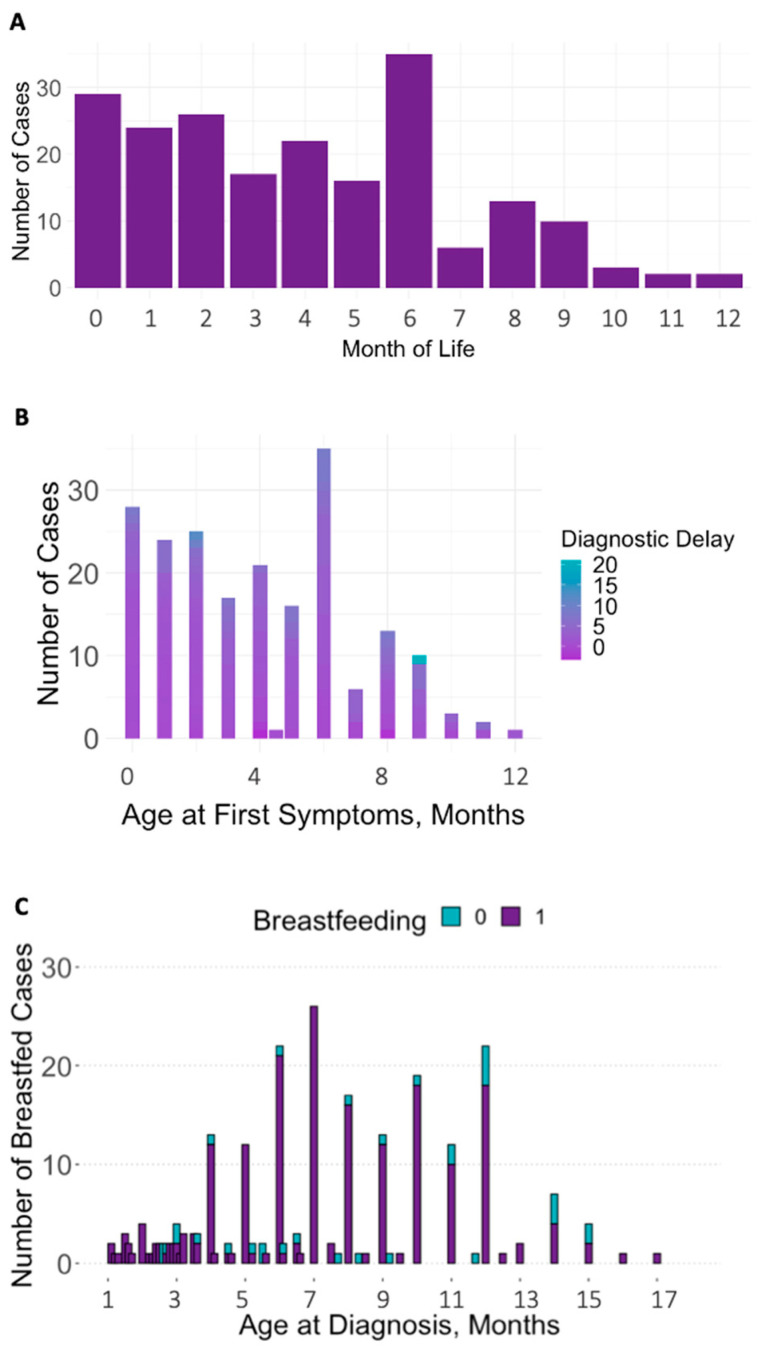
(**A**–**C**) Number of cases with first symptoms per month of life (**A**), diagnostic delay by age at first symptoms (**B**), and exclusive breastfeeding by age at diagnosis (**C**).

The 102 publications [30,31,32,33,34,35,36,37,38,39,40,41,42,43,44,45,46,47,48,49,50,51,52,53,54,55,56,57,58,59,60,61,62,63,64,65,66,67,68,69,70,71,72,73,74,75,76,77,78,79,80,81,82,83,84,85,86,87,88,89,90,91,92,93,94,95,96,97,98,99,100,101,102,103,104,105,106,107,108,109,110,111,112,113,114,115,116,117,118,119,120,121,122,123,124,125,126,127,128,129,130,131] involved 292 infants (189 males). Descriptive data on medical information (age at symptom onset and age at diagnosis), biomarkers, clinical outcomes, and diet are depicted in Table 1 and Figure 2 and Figure 3. Due to counts below the recommended sample size of 80 for regression analyses with four covariates [132], the biochemical parameters of infant MMA in urine and holo-TC, maternal MMA in plasma, holo-TC, tHcy, serum folate, and the clinical signs seizures, solid food refusal, vomiting, somnolence/coma, and maternal B12 malabsorption (intrinsic factor antibodies and parietal cell antibodies) were not included in the further analyses. The mean age at first symptoms was 4 months (0–12), the mean age at diagnosis was 7 months (0–30), and the mean diagnostic delay was 3 months (0–21). The number of cases with first symptoms per month of life, age at symptoms and delay to diagnosis, and rate of exclusive breastfeeding by infant age are depicted in Figure 4. Age at diagnosis was used as a proxy for infant age, as it related to the information on exclusive breastfeeding since this information was mostly recorded at diagnosis and had, most probably, also been the feeding method when the first symptoms occurred. 

### 3.3. Correlation Analyses 

Maternal and infant B12 were positively correlated. Both infant and maternal B12 were negatively correlated with age at first symptoms and age at diagnosis (all *p* < 0.01), indicating that the later in life B12 was measured, the lower the blood levels. Infant plasma MMA and tHcy were negatively correlated with infant and maternal B12 (all *p* < 0.01), underscoring that low B12 values in mother and child are associated with high MMA and tHcy concentrations in the children.

**Figure 5:**
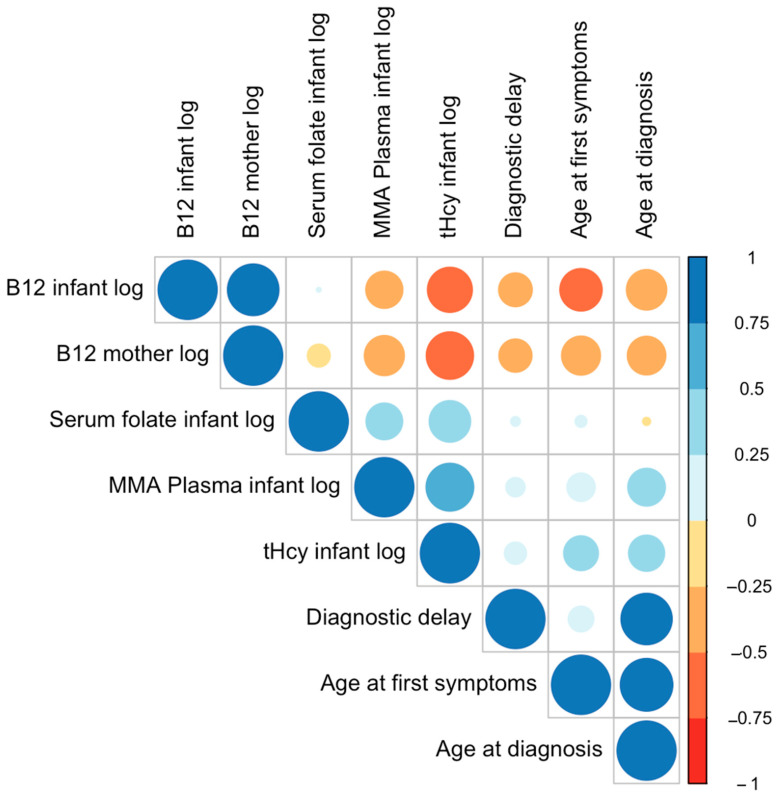
Correlation plot with biomarkers (log), age, and diagnostic delay. Larger circles indicate stronger correlations. The circle color represents the direction of the correlation—warm colors for negative correlations and cool colors for positive correlations.

Infant plasma MMA levels were positively associated with age at first symptoms (*p* < 0.05) and at diagnosis (*p* < 0.01). This direction of effect was also evident for infant tHcy (all *p* < 0.01). MMA and tHcy correlated positively (*p* < 0.01). Infant serum folate was positively correlated with infant plasma MMA and tHcy (all *p* < 0.01) but showed no significant correlation with age at first symptoms, age at diagnosis, diagnostic delay, and infant and maternal B12 levels. Diagnostic delay (calculated from age at diagnosis and first symptoms) correlated positively with both age at first symptoms and age at diagnosis (all *p* < 0.01), indicating that the older the child at first symptoms, the longer the delay to diagnosis (Table 2, Figure 5).

### 3.4. Association between Clinical Symptoms: Chi-Squared Tests

Infants with anemia significantly more often showed irritability, hypotonia, and apathy/lethargy compared to infants not presenting with anemia (see Table 3). 

Infants with apathy/lethargy had significantly more irritability, developmental delay, and movement disorders than infants without apathy/lethargy. Chi-squared tests for failure to thrive and cerebral atrophy did not reach a level of significance.

### 3.5. Subsample Comparisons: Descriptive Statistics, Chi-Squared Tests, and ANOVAs 

The results of chi-squared tests comparing B12 groups concerning clinical outcomes and dietary variables are presented in Table 4.

Chi-squared tests show that irritability, anemia, hypotonia, apathy/lethargy, and movement disorders were significantly more frequent among infants with probable B12 deficiency than in the other two groups.

Exclusive breastfeeding was more frequent in children with probable B12 deficiency. 

More mothers of infants with only possible or even unlikely B12 deficiency followed a mixed diet, and none of the mothers followed a vegan, vegetarian, or mostly vegetarian diet. 

ANOVAs (Table 5)showed that mean maternal B12 levels were highest for infants with unlikely B12 deficiency, followed by the possible and probable deficiency groups. The mean ages at onset of first symptoms and at diagnosis were highest for the probable B12-deficiency group, followed by the possible and the unlikely B12-deficiency groups.

Infants in the latter group were diagnosed as B12 deficient based on elevated plasma MMA (n = 17) and/or plasma tHcy concentrations (n = 15), or clinically by resolution of symptoms under B12 treatment (n = 3).

### 3.6. Regression Analysis—Predicting Infant Biomarkers

Higher maternal B12 levels and a mixed maternal diet predicted higher infant B12.

Exclusive breastfeeding, higher age at first symptoms or at diagnosis, and longer delay to diagnosis predicted lower infant B12 levels. Higher maternal B12 levels with a small effect size and a mixed diet with a very large effect size were both predictive of lower infant tHcy levels (Table 6). No significant relationships were found for infant MMA in plasma. 

### 3.7. Regression Analysis—Predicting Clinical Outcomes

Lower odds for anemia were predicted by higher maternal (OR = 0.08, *p* < 0.001) and infant B12 (OR = 0.07, *p* < 0.001). Higher odds for anemia were predicted by higher infant tHcy levels (OR = 4.42, *p* < 0.05), being older at first symptoms (OR = 1.38, *p* < 0.05), and longer diagnostic delay (OR = 1.38, *p* < 0.05).

Higher maternal (OR = 0.07, *p* < 0.05) and infant B12 (OR = 0.02, *p* < 0.05) predicted lower odds for apathy/lethargy. Exclusive breastfeeding (OR = 22.65, *p* < 0.05) and longer diagnostic delay predicted significantly higher odds of apathy/lethargy (OR = 1.26, *p* < 0.05). Higher maternal B12 predicted lower odds for movement disorder (OR = 0.06, *p* < 0.01).

No significant predictors were found for failure to thrive, irritability, cerebral atrophy, hypotonia, and developmental delay. 

### 3.8. Regression Analysis—Infant Biomarkers and Medical Data as Moderators 

No significant interactions nor mediations were detectable when diet variables and medical data were used as moderators and mediators for the relationship between infant biomarkers and clinical outcomes. Furthermore, there was no interaction between maternal diet and supplement intake concerning clinical outcomes. A significant interaction was present between the age at first symptoms and exclusive breastfeeding, in that exclusively breastfed infants had higher odds for apathy/lethargy when age at symptom onset was higher.

## 4. Discussion

The 292 cases with a clinically established diagnosis of B12 deficiency included in our analysis were published over a period of 60 years and showed a significant diagnostic delay in many cases, indicating that efforts to prevent this serious condition are not yet successful overall.

We aimed to describe the clinical presentation of B12 deficiency to delineate ways for early, effective diagnosis and screening or prevention. Although our study included pooled data that represents the largest sample of infants with clinically diagnosed B12 deficiency, there are some limitations. Attempts to obtain missing data by requesting it from the authors were met with limited success. Missing values resulting in small sample sizes for some parameters did not allow for subsample analyses across all biomarkers and clinical data. The high proportion of subjects that were exclusively breastfed caused an uneven representation of nutrition forms, which may have implications for the statistical power of the analyses. Another limitation is the heterogeneity between included studies regarding measurement assays and cut-off values for biomarkers. To address this concern, all numbers were standardized, and guidelines were utilized to classify values. As in any study relying on published data, the quality, accuracy, and completeness of the data depend on the methods and the reporting standards (including publication bias) of the primary studies. The populations, settings, and methods of the included studies may not be fully representative of the population of infants with B12 deficiency, and it cannot completely be excluded for all the reports that the diagnosis of B12 deficiency was correct and that rare inborn errors of B12 absorption and trafficking have not been overlooked. Furthermore, any retrospective analysis of cross-sectional data is limited to exploring correlational rather than causal relationships.

Clinical symptoms of infant B12 deficiency occur in most cases within the first six months of life. An infant’s clinical pattern that alerts toward B12 deficiency is anemia accompanied by a cluster of neurological symptoms [128], predominantly irritability, apathy or lethargy, hypotonia, and movement disorders. Taking the dietary history of the mother (and child) is essential; most children in the analyzed sample were exclusively breastfed, and our data support the observation that a maternal vegan or vegetarian diet enhances the risk for B12 deficiency [133]. B12 malabsorption was diagnosed in 52 cases (four supplemented). Thirty-five mothers were on B12 supplements without suffering from B12 malabsorption. No precise information was available on how long and in what dosage B12 supplements were taken in either group. It has been shown that small doses of B12, up to 3 μg/day during pregnancy, seem not to prevent B12 deficiency effectively [134]. Accordingly, women must be carefully monitored for their B12 status during pregnancy and lactation, even if on supplements. 

The biochemical diagnosis of B12 deficiency is still challenging. While some recommendations rely on variable B12 cutoffs [10,14,15,16], others imply that very mildly elevated tHcy concentrations, such as ≥6.5 μmol/L [135] or >8 μmol/L [136], indicate B12 deficiency, even if B12 concentration is normal [137]. The approach of establishing 95% reference intervals for B12 (180–1400 pmol/L) for infants below one year based on Canadian [138] and Danish populations [139] seems promising and could be pursued for other populations too. However, 69 of the analyzed cases had B12 concentrations within the 95% reference intervals [139], which confirms the statement that severe functional and clinically relevant B12 deficiency may exist even with normal B12 concentrations, according to present definitions [10]. Accordingly, physicians based their diagnosis of B12 deficiency on elevated tHcy and/or MMA in most of the 19 cases, with B12 concentrations above 258 pmol/L in the studied publications. 

MMA and tHcy were measured in growing numbers in more recent publications. MMA and tHcy indicate the intracellular availability of B12 and are considered to detect B12 deficiency with higher sensitivity [10]. Our analyses prove that infant and maternal B12 concentrations at diagnosis of B12 deficiency are highly correlated. The lower the B12, the higher the closely interrelated parameters of infant MMA and tHcy. Our data do not provide additional insights on whether tHcy in early life is more sensitive than MMA in detecting B12 deficiency [140]. As outlined above, cutoffs for tHcy indicating relevant B12 deficiency are not yet standardized and the cutoffs for plasma/serum MMA are also under discussion. Thirty-one of the analyzed cases had MMA concentrations within the recently suggested 95% reference interval of 0.1 to 1.25 µmol/L [139]. We assume that the most sensible approach to diagnosis lies in the joint analysis of B12, tHcy, and MMA. 

Holo-TC, the transcobalamin-bound B12 fraction able to enter the cells, has only recently been introduced as an additional informative parameter, but age-adapted norms for holoTC have not yet been widely established [10]. Holo-TC was rarely measured, and none of the reported cases had been diagnosed based on their holoTC levels. 

Maternal B12 concentrations during pregnancy are correlated with infant B12 concentrations at birth and at age 6 months [135]. The persistence of the close relation between maternal and infant values beyond pregnancy could be confirmed in our analyses, because, even at the time of diagnosis with clinically evident infant B12 deficiency, maternal and infant B12 concentrations were correlated and predicted infant MMA and tHcy concentrations, as well as the clinical signs anemia, apathy/lethargy, and movement disorders. 

While elevated MMA and tHcy are valuable indicators for B12 deficiency, their concentrations did not correlate with clinical symptoms. Inborn errors of B12 metabolism affecting both tHcy and MMA metabolism, such as the cobalamin C defect, have a wide spectrum of clinical presentations from severe, neonatal disease to attenuated, late manifesting cases. The biomarkers tHcy and MMA, however, are equally elevated in severe and attenuated cases. However, late-onset cases diagnosed later and exposed longer to elevated tHcy and MMA present with less severe disease [141], suggesting that tHcy and MMA may not be the main pathophysiological culprits. Accordingly, MMA and tHcy should be understood as diagnostic parameters for B12 deficiency rather than as numeric predictors of clinical affections or severity. 

Maternal and infant B12 concentrations were lower in infants that were older at diagnosis, suggesting that, under certain circumstances, B12 deficiency aggravates with time. Neonatal B12 stores are low if the mother is deficient, and with exclusive breastfeeding, maternal, and infant B12 concentrations decline further [33]. In the analyzed cases, exclusive breastfeeding was associated with lower infant B12 concentrations, and, and due to its occurrence in 86% of cases, breastfeeding was the most frequent nutrition. A state of infant B12 deficiency may facilitate prolonged exclusive breastfeeding because a refusal of solid foods and difficulties in weaning are often observed in infants with B12 deficiency [44]. 

It is known that infants on formula are less likely to develop B12 deficiency [142]. However, it is of utmost importance to highlight that our results do not weaken the worldwide encouragement of breastfeeding because its overwhelmingly beneficial effects are beyond doubt [143]. It must, however, be ensured that mothers can breastfeed safely in terms of B12 supply. 

It has been observed that low maternal serum B12 in the first three months of pregnancy correlates with high infant MMA-related markers in newborn screening (NBS) [134]. It seems, therefore, of the utmost importance to identify women with a B12 deficiency as early as possible during or even before pregnancy. 

Presently, suspected neonatal B12 deficiency is a frequent cause for recalls from NBS programs targeting inborn errors of B12 metabolism or MMAurias. Some authors advise using NBS as the strategy of choice to identify B12-deficient children [144,145]. It has, however, been shown that children who become symptomatic with severe B12 deficiency during infancy and, as such, a group that NBS should detect, are mostly not identified [146,147]. NBS may thus not be the optimal strategy to prevent severe B12 deficiency and its devastating impact. 

The exact benefits of the cohort with suboptimal B12 status from being detected by NBS are unknown. The quality of the evidence to associate very subtle B12 deficiency with impaired cognitive development in infants and children is suboptimal. Studies are methodologically diverse and have been conducted in different populations; their results are conflicting [136,148,149,150]. Considering that any NBS recall is a major event for new parents with a significant impact on the attitude towards their child, even if the recall is “harmless” [151] and that the workup of a neonate with suspected B12 deficiency triggers invasive and costly diagnostic steps, the benefits of NBS for B12 deficiency must be thoroughly evaluated. 

Counseling of women during pregnancy, including careful assessment of maternal diet and history-taking regarding diseases causing B12 deficiency and maternal gastric surgery, probably is a less invasive and widely applicable approach. 

For the prevention of neural tube defects, flour is fortified with folic acid in the United States, while in Europe, folic acid supplementation is recommended for women planning to conceive, and approximately 80% of women adhere to this recommendation [152]. A similarly broad campaign recommending B12 supplements during pregnancy and breastfeeding could be considered with all required caution towards potential adverse effects. Such a strategy would, however, fail for women with B12 absorption defects.

An obligatory blood test for B12 during pregnancy would detect more B12-deficient mothers but would still miss cases. It is difficult to estimate the exact increase in sensitivity of the test if additional measurements such as MMA, tHcy, or holo-TC are taken. Healthcare providers and reimbursement stakeholders must thoroughly consider the costs and logistics for this option. With the introduction of such a strategy, doctors must be aware of the specific requirements and cutoff values for B12 during pregnancy.

## 5. Conclusions and Implications for Clinical Practice

Low maternal B12 results in reduced neonatal B12 status aggravated by a low B12 supply in exclusively breastfed infants. Exclusive measurement of B12 blood concentrations may miss cases of clinically relevant B12 deficiency and should be complemented by measurements of MMA and tHcy. The additional value of holo-TC should be explored. Anemia accompanied by neurological symptoms should alert towards B12 deficiency in infants. NBS seems to not be the ideal preventive method to detect severe B12 deficiency, but it detects a different population for which the benefit of detection remains to be clarified. Creating clinical awareness of the condition not only among pediatricians but also among physicians who care for pregnant women, and in the female population of childbearing age, is essential. It is advisable to specifically alert women who adhere to a vegan or vegetarian diet and are planning to get pregnant to have their B12 status examined. 

## Figures and Tables

**Table 1:** Sample Characteristics.

Variable	Total Sample (*N* = 292) (% Values Relate to the Number of Reports for Each Variable)
Sex female, n (%)	103 (37)
Age at first symptoms (months), M (SD), range	4 (3), 0–12
Age at diagnosis (months), M (SD), range	7 (4), 0–30
Diagnostic delay (months), M (SD), range	3 (3), 0-21
Serum B12 infant log, M (SD), range	5 (1), 1–7
Serum B12 infant pmol/L, M (SD), range	107 (87), 2–552
^#^ Probable B12 deficiency, n (%)	204 (78)
^#^ Possible B12 deficiency, n (%)	38 (15)
^#^ Unlikely B12 deficiency, n (%)	19 (7)
Plasma MMA infant log, M (SD), range	1 (1.35), −2–4
Plasma MMA infant µmol/L, M (SD), range	5 (9), 0–46
Plasma tHcy infant log, M (SD), range	3 (1), 0–5
Plasma tHcy infant µmol/L, M (SD), range	51 (56), 1–239
Serum folate infant log, M (SD), range	7 (1), −1–5
Serum folate infant nmol/L, M (SD), range	36 (21), 0–211
Serum B12 mother log, M (SD), range	5 (1), 3–7
Serum B12 mother pmol/L, M (SD), range	154 (109), 10–709
Clinical symptoms: Failure to thrive, n (%)	89 (86)
Irritability, n (%)	38 (43)
Anemia, n (%)	186 (69)
Cerebral Atrophy, n (%)	58 (72)
Hypotonia, n (%)	165 (96)
Apathy/lethargy, n (%)	86 (61)
Developmental delay, n (%)	146 (93)
Movement disorder, n (%)	74 (56)
Maternal Diet: Vegan, n (%)	35 (17)
Vegetarian, n (%)	29 (14)
Mostly vegetarian, n (%)	16 (8)
Mixed, n (%)	126 (61)
Maternal B12 malabsorption (with supplementation), n (n)	52 (4)
Maternal B12 supplementation at diagnosis without malabsorption, n (%)	35 (38)
Diet of the infant: Exclusive breastfeeding, n (%)	224 (86)
Partial breastfeeding, n (%)	12 (7)
Partial formula, n (%)	3 (7)
Solid foods vegetarian, n (%)	2 (1)
Solid foods vegan, n (%)	3 (2)
Solid foods mixed, n	0

MMA: methylmalonic acid, tHcy: total homocysteine, M: mean, SD: standard deviation. All percentages refer to the total number of entries. ^#^ [14,15,16].

**Table 2:** Means, standard deviations, and correlations with confidence intervals for biomarkers (log), age, and diagnostic delay.

Variable	M	SD	Age at First Symptoms (Months)	Age at Diagnosis (Months)	Diagnostic Delay (Months)	B12 Infant Log	MMA Plasma Infant Log	tHcy Infant Log	Serum Folate Infant Log
Age at first symptoms	3.99	2.97							
Age at diagnosis	7.14	4.03	0.79 ** [0.73, 0.84]						
Diagnostic delay	2.58	2.72	0.19 ** [0.05, 0.32]	0.75 ** [0.68, 0.81]					
B12 infant log	4.67	0.81	−0.51 ** [−0.61, −0.40]	−0.46 ** [−0.56, −0.36]	−0.32 ** [−0.45, −0.19]				
MMA Plasma infant log	0.66	1.35	0.23 * [0.01, 0.43]	0.41 ** [0.21, 0.57]	0.11 [−0.12, 0.32]	−0.39 ** [−0.57, −0.18]			
tHcy infant log	3.31	1.14	0.34 ** [0.18, 0.49]	0.37 ** [0.23, 0.50]	0.14 [−0.03, 0.31]	−0.58 ** [−0.68, −0.45]	0.65 ** [0.50, 0.76]		
Serum folate infant log	2.66	0.58	0.04 [−0.14, 0.22]	−0.02 [−0.19, 0.15]	0.03 [−0.16, 0.21]	0.01 [−0.17, 0.18]	0.38 ** [0.15, 0.58]	0.49 ** [0.31, 0.64]	
B12 mother log	5.14	0.65	−0.43 ** [−0.55, −0.30]	−0.42 ** [−0.52, −0.31]	−0.31 ** [−0.44, −0.17]	0.76 ** [0.70, 0.81]	−0.46 ** [−0.62, −0.025]	−0.64 ** [−0.73, −0.53]	−0.15 [−0.33, 0.04]

M: mean, SD: standard deviation. Values in square brackets: 95% confidence interval. * *p* < 0.05. ** *p* < 0.01.

**Table 3:** Chi-squared tests and frequencies: clinical symptoms.

Count Variable	n		Chi-Squared	*p*
	Anemia	No Anemia		
Irritability	24	12	14	<0.001
Hypotonia	108	40	8	<0.01
Apathy/lethargy	75	8	52	<0.001
	**Apathy/lethargy**	**No apathy/lethargy**		
Irritability	16	5	23	<0.001
Developmental delay	69	1	5	<0.05
Movement disorders	29	12	12	<0.001

n: sample size, *p*: *p*-value.

**Table 4:** Chi-squared tests and frequencies: subgroups of infants with probable, possible, and unlikely B12 deficiency.

Count Variable	n			Chi-Squared	*p*
	Probable B12 Deficiency	Possible B12 Deficiency	Unlikely B12 Deficiency		
Irritability	25	4	4	19	<0.001
Anemia	149	9	2	69	<0.001
Hypotonia	122	12	11	9	<0.05
Apathy/lethargy	37	1	1	60	<0.001
Movement disorder	46	13	6	7	<0.05
Exclusive breastfeeding	158	26	13	13	<0.05
Vegan diet Mixed diet	25	0	0	41	<0.001
Vegetarian diet	27	0	0		
Mixed diet	64	35	17		
Mostly vegetarian diet	14	0	0		

n: sample size, *p*: *p*-value.

**Table 5:** Fixed-effects ANOVAs and means.

Predictor		M		F	*p*
	Probable Deficiency	Possible Deficiency	Unlikely Deficiency		
A. Criterion: maternal B12 levels					
(Intercept)				760	<0.001
B12 infant grouped	163	385	421	80	<0.001
B. Criterion: age at diagnosis					
(Intercept)				192	<0.001
B12 infant grouped	8	4	3	39	<0.001
C. Criterion: age at first symptoms					
(Intercept)				106	<0.001
B12 infant grouped	5	2	1	27	<0.001

M = mean, F = F-test, *p* = *p*-value.

**Table 6:** Regression results with infant B12 log (A) and infant tHcy log (B) as the criteria.

**A** **Predictor**	**b**	**b** **95% CI** **[LL, UL]**
(Intercept)	0.65	[−0.90, 2.20]
Maternal B12, log	0.79 **	[0.55, 1.03]
Sex female	0.11	[−0.13, 0.36]
Age at first symptoms	−0.03	[−0.09, 0.04]
Age at diagnosis	−0.04	[−0.08, 0.01]
Serum folate infant, log	0.11	[−0.09, 0.31]
(Intercept, reference category vegan)	4.52 **	[3.48, 5.56]
Maternal diet vegetarian	0.42	[−0.14, 0.99]
Maternal mixed	0.72 **	[0.23, 1.22]
Maternal mostly vegetarian	0.36	[−0.57, 1.28]
Sex female	0.08	[−0.18, 0.33]
Age at first symptoms	−0.06	[−0.13, 0.02]
Age at diagnosis	−0.06 *	[−0.11, −0.00]
Serum folate infant, log	0.14	[−0.22, 0.50]
(Intercept)	5.95 **	[5.27, 6.63]
Breastfeeding exclusively	−0.58 **	[−0.88, −0.27]
Sex female	0.10	[−0.14, 0.35]
Age at first symptoms	−0.05	[−0.12, 0.02]
Age at diagnosis	−0.10 **	[−0.15, −0.05]
Serum folate infant, log	0.07	[−0.14, 0.28]
(Intercept)	5.36 **	[4.74, 5.99]
Age at first symptoms	−0.08 *	[−0.15, −0.01]
Age at diagnosis	−0.07 **	[−0.12, −0.02]
Sex female	0.19	[−0.07, 0.44]
Serum folate infant, log	0.08	[−0.14, 0.30]
(Intercept)	5.36 **	[4.74, 5.99]
Age at diagnosis	−0.07 **	[−0.12, −0.02]
Age at first symptoms	−0.08 *	[−0.15, −0.01]
Sex female	0.19	[−0.07, 0.44]
Serum folate infant, log	0.08	[−0.14, 0.30]
(Intercept)	5.03 **	[4.30, 5.77]
Diagnostic delay	−0.09 **	[−0.15, −0.04]
Sex female	0.22	[−0.08, 0.52]
Serum folate infant, log	0.06	[−0.20, 0.32]
**B** **Predictor**	**b**	**b** **95% CI** **[LL, UL]**
(Intercept)	3.40 *	[0.76, 6.04]
Maternal B12, log	−0.51 **	[−0.86, −0.16]
Sex female	0.11	[−0.22, 0.44]
Age at first symptoms	−0.05	[−0.15, 0.05]
Age at diagnosis	0.02	[−0.05, 0.09]
Serum folate infant, log	0.83 **	[0.35, 1.31]
(Intercept, reference category vegan)	1.76 **	[0.44, 3.07]
Maternal diet vegetarian	−0.55	[−1.70, 0.60]
Maternal mixed	−1.00 *	[−1.81, −0.20]
Maternal mostly vegetarian	0.53	[−0.85, 1.91]
Sex female	0.18	[−0.09, 0.44]
Age at first symptoms	−0.02	[−0.10, 0.07]
Age at diagnosis	0.01	[−0.05, 0.07]
Serum folate infant, log	0.66 **	[0.24, 1.09]

A significant b weight indicates the semipartial correlation is also significant. b: unstandardized regression weights. LL: lower limit, UL: upper limit of a confidence interval. * *p* < 0.05, ** *p* < 0.01.

## Data Availability

Access to the data included in the analyses on request to the corresponding author.

## Abbreviations

B12	Vitamin B12
Cbl	Cobalamin
Hcy	Homocysteine
tHcy	Total Homocysteine
MMA	Methylmalonic acid
HoloTC	Holo-Transcobalamin
NBS	Newborn Screening

## References

[1] de Souza A., Moloi M.W. (2014). Involuntary movements due to vitamin B_12_ deficiency. Neurol. Res..

[2] Dror D.K., Allen L.H. (2008). Effect of vitamin B_12_ deficiency on neurodevelopment in infants: Current knowledge and possible mechanisms. Nutr. Rev..

[3] Huemer M., Baumgartner M.R. (2019). The clinical presentation of cobalamin-related disorders: From acquired deficiencies to inborn errors of absorption and intracellular pathways. J. Inherit. Metab. Dis..

[4] Ströhle A., Richter M., González-Gross M., Neuhäuser-Berthold M., Wagner K.H., Leschik-Bonnet E., Egert S. (2019). German Nutrition Society (DGE). The Revised D-A-CH-Reference Values for the Intake of Vitamin B_12_: Prevention of Deficiency and Beyond. Mol. Nutr. Food Res..

[5] Rashid S., Meier V., Patrick H. (2021). Review of Vitamin B_12_ deficiency in pregnancy: A diagnosis not to miss as veganism and vegetarianism become more prevalent. Eur. J. Haematol..

[6] Obeid R., Morkbak A.L., Munz W., Nexo E., Herrmann W. (2006). The cobalamin-binding proteins transcobalamin and haptocorrin in maternal and cord blood sera at birth. Clin. Chem..

[7] Layden A.J., O’Brien K.O., Pressman E.K., Cooper E.M., Kent T.R., Finkelstein J.L. (2016). Vitamin B_12_ and placental expression of transcobalamin in pregnant adolescents. Placenta.

[8] Duggan C., Srinivasan K., Thomas T., Samuel T., Rajendran R., Muthayya S., Finkelstein J.L., Lukose A., Fawzi W., Allen L.H. (2014). Vitamin B-12 supplementation during pregnancy and early lactation increases maternal, breast milk, and infant measures of vitamin B-12 status. J. Nutr..

[9] Hay G., Johnston C., Whitelaw A., Trygg K., Refsum H. (2008). Folate and cobalamin status in relation to breastfeeding and weaning in healthy infants. Am. J. Clin. Nutr..

[10] Hannibal L., Lysne V., Bjørke-Monsen A.L., Behringer S., Grünert S.C., Spiekerkoetter U., Jacobsen D.W., Blom H.J. (2016). Biomarkers and Algorithms for the Diagnosis of Vitamin B_12_ Deficiency. Front. Mol. Biosci..

[11] Page M.J., Moher D., Bossuyt P.M., Boutron I., Hoffmann T.C., Mulrow C.D., Shamseer L., Tetzlaff J.M., Akl E.A., Brennan S.E. (2021). The PRISMA 2020 statement: An updated guideline for reporting systematic reviews. Bmj.

[12] Moola S., Munn Z., Tufanaru C., Aromataris E., Sears K., Sfetcu R., Currie M., Lisy K., Qureshi R., Mattis P., Aromataris E., Munn Z. (2020). Chapter 7: Systematic reviews of etiology and risk. JBI Manual for Evidence Synthesis.

[13] Team R (2020). RStudio: Integrated Development Environment for R RStudio, PBC, Boston, MA. http://www.rstudio.com/.

[14] Snow C.F. (1999). Laboratory diagnosis of vitamin B_12_ and folate deficiency: A guide for the primary care physician. Arch. Intern. Med..

[15] Devalia V., Hamilton M.S., Molloy A.M. (2014). Guidelines for the diagnosis and treatment of cobalamin and folate disorders. Br. J. Haematol..

[16] https://bestpractice.bmj.com/topics/en-gb/822?bcgovtm=pique%20newsmagazine.

[17] Roumeliotis N., Dix D., Lipson A. (2012). Vitamin B_12_ deficiency in infants secondary to maternal causes. Can. Med. Assoc. J..

[18] von Schenck U., Bender-Gotze C., Koletzko B. (1997). Persistence of neurological damage induced by dietary vitamin B-12 deficiency in infancy. Arch. Dis. Child..

[19] Wong S., Ahmad N., Rossetti A.L. (2022). Vomiting as a Presenting Symptom of Infantile Vitamin B_12_ Deficiency. Cureus.

[20] Feraco P., Incandela F., Franceschi R., Gagliardo C., Bellizzi M. (2021). Clinical and Brain Imaging Findings in a Child with Vitamin B_12_ Deficiency. Pediatr. Rep..

[21] Kaninde A., Katre M., Papadopoulou K., Ramaswamy R. (2021). 55 Regression of milestones in an infant as presenting feature of Maternal Pernicious anaemia. Arch. Dis. Child..

[22] Subramani P., Saranya C.G., Chand G.M., Narayani R.S., James S., Vinoth P.N. (2015). Neuroregression in an infant: A rare cause. S. Afr. J. Child Health.

[23] Delbet J.D., Ulinski T. (2017). Thrombotic microangiopathy and breastfeeding: Where is the link? Questions. Pediatr. Nephrol..

[24] Sklar R. (1986). Nutritional Vitamin B_12_ Deficiency in a Breast-Fed Infant of a Vegan-Diet Mother. Clin. Pediatr..

[25] Sadowitz P.D., Livingston A., Cavanaugh R.M. (1986). Developmental Regression as an Early Manifestation of Vitamin B_12_ Deficiency. Clin. Pediatr..

[26] Chalouhi C., Faesch S., Anthoine-Milhomme M.C., Fulla Y., Dulac O., Chéron G. (2008). Neurological Consequences of Vitamin B_12_ Deficiency and Its Treatment. Pediatr. Emerg. Care.

[27] Lampkin B.C., Shore N.A., Chadwick D. (1966). Megaloblastic Anemia of Infancy Secondary to Maternal Pernicious Anemia. N. Engl. J. Med..

[28] Almadan M.S., Al Awamy B.H., Al Mulhim I.A. (1993). Nutritional Vitamin B_12_ deficiency in infancy. Indian J. Pediatr..

[29] Zetterström R., Franzén S. (1954). Megaloblastic Anemia in Infancy: Megaloblastic Anemia Occurring in an Infant of a Mother Suffering from Pernicious Anemia of Pregnancy. Acta Paediatr..

[30] Schlapbach L.J., Schütz B., Nuoffer J.M., Brekenfeld C., Müller G., Fluri S. (2007). Floppy baby with macrocytic anemia and vegan mother. Praxis.

[31] Akcaboy M., Malbora B., Zorlu P., Altınel E., Oguz M.M., Senel S. (2015). Vitamin B_12_ Deficiency in Infants. Indian J. Pediatr..

[32] Sturm I., Hennermann J.B., von Arnim-Baas A., Driever P.H., Massenkeil G. (2008). Thromboembolic events, abortions and a sick infant—Unusual presentation of a vitamin deficiency. Internist.

[33] Hasbaoui B.E., Mebrouk N., Saghir S., Yajouri A.E., Abilkassem R., Agadr A. (2021). Vitamin B_12_ deficiency: Case report and review of literature. Pan. Afr. Med. J..

[34] Sharma N., Kunwar S., Shrestha A.K. (2021). Vitamin B_12_ Deficiency Resembling Acute Leukemia: A Case Report. JNMA J. Nepal Med. Assoc..

[35] Quentin C., Huybrechts S., Rozen L., De Laet C., Demulder A., Ferster A. (2012). Vitamin B_12_ deficiency in a 9-month-old boy. Eur. J. Pediatr..

[36] Roschitz B., Plecko B., Huemer M., Biebl A., Foerster H., Sperl W. (2005). Nutritional infantile vitamin B_12_ deficiency: Pathobiochemical considerations in seven patients. Arch. Dis. Child. Fetal Neonatal Ed..

[37] Dubaj C., Czyż K., Furmaga-Jabłońska W. (2020). Vitamin B_12_ deficiency as a cause of severe neurological symptoms in breast fed infant—A case report. Ital. J. Pediatr..

[38] Guez S., Chiarelli G., Menni F., Salera S., Principi N., Esposito S. (2012). Severe vitamin B_12_ deficiency in an exclusively breastfed 5-month-old Italian infant born to a mother receiving multivitamin supplementation during pregnancy. BMC Pediatr..

[39] Tan M.L., Goh M.C., Fu K.X., Aw M.H., Quak S.H., Goh D.L. (2015). Severe vitamin B_12_ deficiency in a 7-month-old boy. Ann. Acad. Med. Singap..

[40] Erol I., Alehan F., Gümüs A. (2007). West syndrome in an infant with vitamin B_12_ deficiency in the absence of macrocytic anaemia. Dev. Med. Child Neurol..

[41] McPhee A.J., Davidson G.P., Leahy M., Beare T. (1988). Vitamin B_12_ deficiency in a breast fed infant. Arch. Dis. Child..

[42] Kocaoglu C., Akin F., Caksen H., Böke S.B., Arslan S., Aygün S. (2014). Cerebral atrophy in a vitamin B_12_-deficient infant of a vegetarian mother. J. Health Popul. Nutr..

[43] Afzal T., Ashraf N., Munir S., Tabassum R. (2020). Megaloblastic anaemia in a 9-weeks old infant: A case report. J. Pak. Med. Assoc..

[44] Ide E., Van Biervliet S., Thijs J., Vande Velde S., De Bruyne R., Van Winckel M. (2011). Solid food refusal as the presenting sign of vitamin B_12_ deficiency in a breastfed infant. Eur. J. Pediatr..

[45] Korenke G.C., Hunneman D.H., Eber S., Hanefeld F. (2004). Severe encephalopathy with epilepsy in an infant caused by subclinical maternal pernicious anaemia: Case report and review of the literature. Eur. J. Pediatr..

[46] Hoey H., Linnell J.C., Oberholzer V.G., Laurance B.M. (1982). Vitamin B_12_ deficiency in a breastfed infant of a mother with pernicious anaemia. J. R. Soc. Med..

[47] Rodrigues V., Dias A., Brito M.J., Galvão I., Ferreira G.C. (2011). Severe megaloblastic anaemia in an infant. BMJ Case Rep..

[48] Baatenburg de Jong R., Bekhof J., Roorda R., Zwart P. (2005). Severe nutritional vitamin deficiency in a breast-fed infant of a vegan mother. Eur. J. Pediatr..

[49] Rachmel A., Steinberg T., Ashkenazi S., Sela B.A. (2003). Cobalamin deficiency in a breast-fed infant of a vegetarian mother. Isr. Med. Assoc. J..

[50] McNeil K., Chowdhury D., Penney L., Rashid M. (2014). Vitamin B_12_ deficiency with intrinsic factor antibodies in an infant with poor growth and developmental delay. Paediatr. Child Health.

[51] Serin H.M., Kara A.O., Oğuz B. (2015). West syndrome due to vitamin B_12_ deficiency. Turk Pediatri Ars..

[52] Agrawal S., Nathani S. (2009). Neuro-regression in vitamin B_12_ deficiency. BMJ Case Rep..

[53] Chong P.F., Matsukura M., Fukui K., Watanabe Y., Matsumoto N., Kira R. (2019). West Syndrome in an Infant with Vitamin B_12_ Deficiency Born to Autoantibodies Positive Mother. Front. Pediatr..

[54] Tamura A., Nino N., Yamamoto N., Naito A., Matsubara K., Nakatani N., Ichikawa T., Nakamura S., Saito A., Kozaki A. (2019). Vitamin B_12_ deficiency anemia in an exclusively breastfed infant born to an ileum-resected mother. Pediatr. Neonatol..

[55] Lücke T., Korenke G.C., Poggenburg I., Bentele K.H., Das A.M., Hartmann H. (2007). Maternal vitamin B_12_ deficiency: Cause for neurological symptoms in infancy. Z. Geburtshilfe Neonatol..

[56] Glaser K., Girschick H.J., Schropp C., Speer C.P. (2015). Psychomotor development following early treatment of severe infantile vitamin B_12_ deficiency and West syndrome—Is everything fine? A case report and review of literature. Brain Dev..

[57] Casella E.B., Valente M., de Navarro J.M., Kok F. (2005). Vitamin B_12_ deficiency in infancy as a cause of developmental regression. Brain Dev..

[58] Yaramis A. (2020). A variety of abnormal movements in 13 cases with nutritional cobalamin deficiency in infants. Med. Hypotheses.

[59] Taskesen M., Yaramis A., Pirinccioglu A.G., Ekici F. (2012). Cranial magnetic resonance imaging findings of nutritional vitamin B_12_ deficiency in 15 hypotonic infants. Eur. J. Paediatr. Neurol..

[60] Pavone P., Sullo F., Falsaperla R., Greco F., Crespo A., Calvo A., Caraballo R. (2021). Vitamin B_12_ Deficiency and West Syndrome: An Uncommon but Preventable Cause of Neurological Disorder. Report on Three Cases, One of Them with Late Onset during Vitamin B_12_ Treatment. Neuropediatrics.

[61] Malbora B., Yuksel D., Aksoy A., Ozkan M. (2014). Two infants with infantile spasms associated with vitamin B_12_ deficiency. Pediatr. Neurol..

[62] Celiker M.Y., Chawla A. (2009). Congenital B_12_ deficiency following maternal gastric bypass. J. Perinatol..

[63] Okamura J., Miyake Y., Kamei M., Ito Y., Matsubayashi T. (2020). Three infants with megaloblastic anemia caused by maternal vitamin B_12_ deficiency. Pediatr. Int..

[64] Reghu A., Hosdurga S., Sandhu B., Spray C. (2005). Vitamin B_12_ deficiency presenting as oedema in infants of vegetarian mothers. Eur. J. Pediatr..

[65] Citak F.E., Citak E.C. (2011). Severe vitamin B_12_ deficiency in a breast fed infant with pancytopenia. J. Trop. Pediatr..

[66] Bicakci Z. (2015). Growth retardation, general hypotonia, and loss of acquired neuromotor skills in the infants of mothers with cobalamin deficiency and the possible role of succinyl-CoA and glycine in the pathogenesis. Medicine.

[67] Lövblad K., Ramelli G., Remonda L., Nirkko A.C., Ozdoba C., Schroth G. (1997). Retardation of myelination due to dietary vitamin B_12_ deficiency: Cranial MRI findings. Pediatr. Radiol..

[68] Lundgren J., Blennow G. (1999). Vitamin B_12_ deficiency may cause benign familial infantile convulsions: A case report. Acta Paediatr..

[69] Horstmann M., Neumaier-Probst E., Lukacs Z., Steinfeld R., Ullrich K., Kohlschütter A. (2003). Infantile cobalamin deficiency with cerebral lactate accumulation and sustained choline depletion. Neuropediatrics.

[70] Renault F., Verstichel P., Ploussard J.P., Costil J. (1999). Neuropathy in two cobalamin-deficient breast-fed infants of vegetarian mothers. Muscle Nerve.

[71] Dilber B., Eyüboğlu İ. (2022). Cranial Magnetic Resonance Imaging Findings in Hypotonic Infants with Cobalamin Deficiency and Combined Methylmalonic Aciduria and Homocystinuria. Klin. Padiatr..

[72] Grattan-Smith P.J., Wilcken B., Procopis P.G., Wise G.A. (1997). The neurological syndrome of infantile cobalamin deficiency: Developmental regression and involuntary movements. Mov. Disord..

[73] Muhammad R., Fernhoff P., Rasmussen S., Bowman B. (2003). Neurologic impairment in children associated with maternal dietary deficiency of cobalamin—Georgia, 2001. MMWR Morb. Mortal Wkly. Rep..

[74] Jagadish Kumar K., Prudhvi S., Balaji K., Rahul R. (2018). Persistent diarrhea, hemolytic anemia, and splenohepatomegaly due to Vitamin B_12_ deficiency in an infant. J. Appl. Hematol..

[75] Rössler J., Breitenstein S., Havers W. (2001). Megaloblastäre Anämien durch Vitamin-B_12_-Mangel im Kindesalter. Monatsschrift Kinderheilkd..

[76] Belen B., Hismi B.O., Kocak U. (2014). Severe vitamin B_12_ deficiency with pancytopenia, hepatosplenomegaly and leukoerythroblastosis in two Syrian refugee infants: A challenge to differentiate from acute leukaemia. BMJ Case Rep..

[77] Milankov O., Bjelica M., Suvajdžić L., Maksic J., Milankov V., Medić D., Ilić N. (2019). Vitamin B_12_-deficient child of a vegan mother. Food Feed. Res..

[78] Singh G., Le D., Schnabl K., Leaker M.T., Steele M., Sparkes R.L. (2016). Vitamin B_12_ Deficiency in Infancy: The Case for Screening. Pediatr. Blood Cancer.

[79] Avci Z., Turul T., Aysun S., Unal I. (2003). Involuntary movements and magnetic resonance imaging findings in infantile cobalamine (vitamin B_12_) deficiency. Pediatrics.

[80] Emery E.S., Homans A.C., Colletti R.B. (1997). Vitamin B_12_ deficiency: A cause of abnormal movements in infants. Pediatrics.

[81] Siddaraju M.L., Sathyabama K.A. (2014). Vitamin B_12_ Deficiency in an Exclusively Breastfed 7-Month-Old Infant Born to a Vegan Mother. Int. J. Sci. Study.

[82] Johnson P.R., Roloff J.S. (1982). Vitamin B_12_ deficiency in an infant strictly breast-fed by a mother with latent pernicious anemia. J. Pediatr..

[83] Yousif T.I., Shukla P.J., Gallagher S. (2016). Severe Vitamin B_12_ Deficiency; an Unusual Cause of Developmental Regression in Infants. Ir. Med. J..

[84] Serin H.M., Arslan E.A. (2019). Neurological symptoms of vitamin B_12_ deficiency: Analysis of pediatric patients. Acta Clin. Croat..

[85] Rajasekaran V., Sheriff J., Moore H., McCay H., Winstanley M. (2020). Infantile B_12_ deficiency with severe thrombocytopenia-an under-recognised public health problem?. N. Z. Med. J..

[86] Abu-Kishk I., Rachmiel M., Hoffmann C., Lahat E., Eshel G. (2009). Infantile encephalopathy due to vitamin deficiency in industrial countries. Childs Nerv. Syst..

[87] Machado R., Furtado F., Kjöllerström P., Cunha F. (2016). Cutaneous hyperpigmentation and cobalamin deficiency. Br. J. Haematol..

[88] Van Noolen L., Nguyen-Morel M.A., Faure P., Corne C. (2014). Don’t forget methylmalonic acid quantification in symptomatic exclusively breast-fed infants. Eur. J. Clin. Nutr..

[89] Kanra G., Cetin M., Unal S., Haliloglu G., Akça T., Akalan N., Kara A. (2005). Answer to hypotonia: A simple hemogram. J. Child Neurol..

[90] Lund A.M. (2019). Questions about a vegan diet should be included in differential diagnostics of neurologically abnormal infants with failure to thrive. Acta Paediatr..

[91] Mellin-Sanchez L., Sondheimer N. (2018). An Infant Refugee with Anemia and Low Serum Vitamin B_12_. Clin. Chem..

[92] Ozer E.A., Turker M., Bakiler A.R., Yaprak I., Ozturk C. (2001). Involuntary movements in infantile cobalamin deficiency appearing after treatment. Pediatr. Neurol..

[93] Weber-Ferro W., Hertzberg C., Röder H., Timme K., Rossi R. (2011). Intact recovery from early ‘acquired methylmalonic aciduria’ secondary to maternal atrophic gastritis. Acta Paediatr..

[94] Patiroglu T., Unal E., Yildirim S. (2013). Infantile tremor syndrome associated with cobalamin therapy: A case report. Clin. Neurol. Neurosurg..

[95] Dilber B., Reis G.P. (2021). Infantile tremor syndrome secondary to peroral vitamin B_12_ replacement therapy: A report of two cases with myoclonus. Turk. J. Pediatr..

[96] Danielsson L., Enocksson E., Hagenfeldt L., Rasmussen E.B., Tillberg E. (1988). Failure to thrive due to subclinical maternal pernicious anemia. Acta Paediatr. Scand..

[97] Heaton D. (1979). Another case of megaloblastic anemia of infancy due to maternal pernicious anemia. N. Engl. J. Med..

[98] Choudhry V.P. (1972). Vitamin B_12_ deficiency in infancy associated with lactose intolerance. Indian J. Pediatr..

[99] Ozdemir O., Baytan B., Gunes A.M., Okan M. (2010). Involuntary movements during vitamin B_12_ treatment. J. Child Neurol..

[100] Baker S.J., Mathan V.I., Abe K. (1970). Beta-melanocyte stimulating hormone levels in subjects with hyperpigmentation associated with megaloblastic anemia. Blood.

[101] Tosun A., Aral Y.Z., Çeçen E., Aydoğdu A., Çetinkaya Çakmak B. (2011). Involuntary movement in infants during vitamin B_12_ treatment. Turk. J. Haematol..

[102] Tunçer G.O., Köker A., Köker S.A., Aba A., Kara T.T., Coban Y., Akbas Y. (2019). Infantile Tremor Syndrome after Peroral and Intramuscular Vitamin B_12_ Therapy: Two Cases. Klin. Padiatr..

[103] Sharawat I.K., Kasinathan A., Sankhyan N. (2018). Infantile Tremor Syndrome: Response to B_12_ Therapy. J. Pediatr..

[104] Kamoun F., Guirat R., Megdich F., Ben Ameur S., Kallel C., Hachicha M. (2017). Frequent Infections, Hypotonia, and Anemia in a Breastfed Infant. J. Pediatr. Hematol. Oncol..

[105] Banka S., Roberts R., Plews D., Newman W.G. (2010). Early diagnosis and treatment of cobalamin deficiency of infancy owing to occult maternal pernicious anemia. J. Pediatr. Hematol. Oncol..

[106] Higginbottom M.C., Sweetman L., Nyhan W.L. (1978). A syndrome of methylmalonic aciduria, homocystinuria, megaloblastic anemia and neurologic abnormalities in a vitamin B_12_-deficient breast-fed infant of a strict vegetarian. N. Engl. J. Med..

[107] Codazzi D., Sala F., Parini R., Langer M. (2005). Coma and respiratory failure in a child with severe vitamin B_12_ deficiency. Pediatr. Crit. Care Med..

[108] Wighton M.C., Manson J.I., Speed I., Robertson E., Chapman E. (1979). Brain damage in infancy and dietary vitamin B_12_ deficiency. Med. J. Aust..

[109] Stollhoff K., Schulte F.J. (1987). Vitamin B_12_ and brain development. Eur. J. Pediatr..

[110] Doyle J.J., Langevin A.M., Zipursky A. (1989). Nutritional vitamin B_12_ deficiency in infancy: Three case reports and a review of the literature. Pediatr. Hematol. Oncol..

[111] Grange D.K., Finlay J.L. (1994). Nutritional vitamin B_12_ deficiency in a breastfed infant following maternal gastric bypass. Pediatr. Hematol. Oncol..

[112] Yenicesu I. (2008). Pancytopenia due to vitamin B_12_ deficiency in a breast-fed infant. Pediatr. Hematol. Oncol..

[113] Kühne T., Bubl R., Baumgartner R. (1991). Maternal vegan diet causing a serious infantile neurological disorder due to vitamin B_12_ deficiency. Eur. J. Pediatr..

[114] Weiss R., Fogelman Y., Bennett M. (2004). Severe vitamin B_12_ deficiency in an infant associated with a maternal deficiency and a strict vegetarian diet. J. Pediatr. Hematol. Oncol..

[115] Kamei M., Ito Y., Ando N., Awaya T., Yamada T., Nakagawa M., Yamaguchi A., Ohuchi M., Yazaki M., Togari H. (2011). Brain atrophy caused by vitamin B_12_-deficient anemia in an infant. J. Pediatr. Hematol. Oncol..

[116] Lampkin B.C., Saunders E.F. (1969). Nutritional vitamin B_12_ deficiency in an infant. J. Pediatr..

[117] Davis J.R., Goldenring J., Lubin B.H. (1981). Nutritional vitamin B_12_ deficiency in infants. Am. J. Dis. Child..

[118] Graham S.M., Arvela O.M., Wise G.A. (1992). Long-term neurologic consequences of nutritional vitamin B_12_ deficiency in infants. J. Pediatr..

[119] Rendle-Short J., Tiernan J.R., Hawgood S. (1979). Vegan mothers with vitamin B_12_ deficiency. Med. J. Aust..

[120] Wardinsky T.D., Montes R.G., Friederich R.L., Broadhurst R.B., Sinnhuber V., Bartholomew D. (1995). Vitamin B_12_ deficiency associated with low breast-milk vitamin B_12_ concentration in an infant following maternal gastric bypass surgery. Arch. Pediatr. Adolesc. Med..

[121] Bobb-Semple A.A., Lau C.S., Teruya-Feldstein J., Wistinghausen B. (2019). A Rare Cause of Pancytopenia in an Exclusively Breastfed Infant. J. Pediatr. Hematol. Oncol..

[122] Turner R.J., Scott-Jupp R., Kohler J.A. (1999). Infantile megaloblastosis secondary to acquired vitamin B_12_ deficiency. Pediatr. Hematol. Oncol..

[123] Sobczyńska-Malefora A., Ramachandran R., Cregeen D., Green E., Bennett P., Harrington D.J., Lemonde H.A. (2017). An infant and mother with severe B_12_ deficiency: Vitamin B_12_ status assessment should be determined in pregnant women with anaemia. Eur. J. Clin. Nutr..

[124] Bidhuri N., Kumar V., Singh R., Singh D.P., Agarwal S., Nandan D. (2020). Diaphragmatic palsy in a 10-month-old boy with infantile tremor syndrome causing respiratory failure with full response to vitamin B_12_ therapy. Paediatr. Int. Child Health.

[125] Gambon R.C., Lentze M.J., Rossi E. (1986). Megaloblastic anaemia in one of monozygous twins breast fed by their vegetarian mother. Eur. J. Pediatr..

[126] Jadhav M., Webb J.K., Vaishnava S., Baker S.J. (1962). Vitamin B_12_ deficiency in Indian infants. A clinical syndrome. Lancet.

[127] Frader J., Reibman B., Turkewitz D. (1978). Vitamin B_12_ deficiency in strict vegetarians. N. Engl. J. Med..

[128] Honzik T., Adamovicova M., Smolka V., Magner M., Hruba E., Zeman J. (2010). Clinical presentation and metabolic consequences in 40 breastfed infants with nutritional vitamin B_12_ deficiency—What have we learned?. Eur. J. Paediatr. Neurol..

[129] Ljungblad U.W., Astrup H., Mørkrid L., Hager H.B., Lindberg M., Eklund E.A., Bjørke-Monsen A.-L., Rootwelt T., Tangeraas T. (2022). Breastfed Infants with Spells, Tremor, or Irritability: Rule Out Vitamin B_12_ Deficiency. Pediatr. Neurol..

[130] Ganesan S., Thanawala N., Hussain N. (2013). Vitamin B_12_ deficiency: A treatable cause of developmental delay in infancy. J. Paediatr. Child Health.

[131] Saritha K., Nalini B., Anjali R. (2018). B_12_ Deficiency in a Breastfed Infant Due to Maternal B_12_ Deficiency: A Case Report. J. Clin. Diagn. Res..

[132] Gotelli N., Ellison A. (2004). A Primer of Ecological Statistics.

[133] Zeuschner C.L., Hokin B.D., Marsh K.A., Saunders A.V., Reid M.A., Ramsay M.R. (2013). Vitamin B_12_ and vegetarian diets. Med. J. Aust..

[134] Dayaldasani A., Ruiz-Escalera J., Rodríguez-Espinosa M., Rueda I., Pérez-Valero V., Yahyaoui R. (2014). Serum vitamin B_12_ levels during the first trimester of pregnancy correlate with newborn screening markers of vitamin B_12_ deficiency. Int. J. Vitam. Nutr. Res..

[135] Varsi K., Ueland P.M., Torsvik I.K., Bjørke-Monsen A.L. (2018). Maternal Serum Cobalamin at 18 Weeks of Pregnancy Predicts Infant Cobalamin Status at 6 Months-A Prospective, Observational Study. J. Nutr..

[136] Ljungblad U.W., Paulsen H., Mørkrid L., Pettersen R.D., Hager H.B., Lindberg M., Astrup H., Eklund E.A., Bjørke-Monsen A.L., Rootwelt T. (2021). The prevalence and clinical relevance of hyperhomocysteinemia suggesting vitamin B_12_ deficiency in presumed healthy infants. Eur. J. Paediatr. Neurol..

[137] Reischl-Hajiabadi A.T., Garbade S.F., Feyh P., Weiss K.H., Mütze U., Kölker S., Hoffmann G.F., Gramer G. (2022). Maternal Vitamin B_12_ Deficiency Detected by Newborn Screening-Evaluation of Causes and Characteristics. Nutrients.

[138] Bohn M.K., Higgins V., Asgari S., Leung F., Hoffman B., Macri J., Adeli K. (2019). Paediatric reference intervals for 17 Roche cobas 8000 e602 immunoassays in the CALIPER cohort of healthy children and adolescents. Clin. Chem. Lab. Med..

[139] Abildgaard A., Knudsen C.S., Hoejskov C.S., Greibe E., Parkner T. (2022). Reference intervals for plasma vitamin B_12_ and plasma/serum methylmalonic acid in Danish children, adults and elderly. Clin. Chim. Acta.

[140] Green R., Allen L.H., Bjørke-Monsen A.L., Brito A., Guéant J.L., Miller J.W., Molloy A.M., Nexo E., Stabler S., Toh B.H. (2017). Vitamin B_12_ deficiency. Nat. Rev. Dis. Primers.

[141] Huemer M., Diodato D., Martinelli D., Olivieri G., Blom H., Gleich F., Kölker S., Kožich V., Morris A.A., Seifert B. (2019). EHOD consortium. Phenotype, treatment practice and outcome in the cobalamin-dependent remethylation disorders and MTHFR deficiency: Data from the E-HOD registry. J. Inherit. Metab. Dis..

[142] Karademir F., Suleymanoglu S., Ersen A., Aydinoz S., Gultepe M., Meral C., Ozkaya H., Gocmen I. (2007). Vitamin B_12_, folate, homocysteine and urinary methylmalonic acid levels in infants. J. Int. Med. Res..

[143] Victora C.G., Bahl R., Barros A.J., França G.V., Horton S., Krasevec J., Murch S., Sankar M.J., Walker N., Rollins N.C. (2016). Breastfeeding in the 21st century: Epidemiology, mechanisms, and lifelong effect. Lancet.

[144] Rozmarič T., Mitulović G., Konstantopoulou V., Goeschl B., Huemer M., Plecko B., Spenger J., Wortmann S.B., Scholl-Bürgi S., Karall D. (2020). Elevated Homocysteine after Elevated Propionylcarnitine or Low Methionine in Newborn Screening Is Highly Predictive for Low Vitamin B_12_ and Holo-Transcobalamin Levels in Newborns. Diagnostics.

[145] Gramer G., Hoffmann G.F. (2020). Vitamin B_12_ Deficiency in Newborns and their Mothers-Novel Approaches to Early Detection, Treatment and Prevention of a Global Health Issue. Curr. Med. Sci..

[146] Tangeraas T., Ljungblad U.W., Lutvica E., Kristensen E., Rowe A.D., Bjørke-Monsen A.L., Rootwelt-Revheim T., Sæves I., Pettersen R.D. (2023). Vitamin B_12_ Deficiency (Un-) Detected Using Newborn Screening in Norway. Int. J. Neonatal Screen..

[147] Ljungblad U.W., Lindberg M., Eklund E.A., Sæves I., Sagredo C., Bjørke-Monsen A.L., Tangeraas T. (2022). A Retrospective Evaluation of the Predictive Value of Newborn Screening for Vitamin B_12_ Deficiency in Symptomatic Infants below 1 Year of Age. Int. J. Neonatal Screen..

[148] Venkatramanan S., Armata I.E., Strupp B.J., Finkelstein J.L. (2016). Vitamin B-12 and Cognition in Children. Adv. Nutr..

[149] Lai J.S., Mohamad Ayob M.N., Cai S., Quah P.L., Gluckman P.D., Shek L.P., Yap F., Tan K.H., Chong Y.S., Godfrey K.M. (2019). Maternal plasma vitamin B_12_ concentrations during pregnancy and infant cognitive outcomes at 2 years of age. Br. J. Nutr..

[150] Thomas S., Thomas T., Bosch R.J., Ramthal A., Bellinger D.C., Kurpad A.V., Duggan C.P., Srinivasan K. (2019). Effect of Maternal Vitamin B_12_ Supplementation on Cognitive Outcomes in South Indian Children: A Randomized Controlled Clinical Trial. Matern. Child Health J..

[151] Tu S.J., Wei Y.J., Chen B.T., Zhang X.F., Luo C., Dong B.Q. (2023). Effects of a false-positive result in newborn congenital hypothyroidism screening on parents in Guangxi, China. Front. Pediatr..

[152] Oliver E.M., Grimshaw K.E.C., Schoemaker A.A., Keil T., McBride D., Sprikkelman A.B., Ragnarsdottir H.S., Trendelenburg V., Emmanouil E., Reche M. (2014). Dietary habits and supplement use in relation to national pregnancy recommendations: Data from the EuroPrevall birth cohort. Matern. Child Health J..

